# *Toxoplasma gondii *infection and abdominal hernia: evidence of a new association

**DOI:** 10.1186/1756-3305-4-112

**Published:** 2011-06-17

**Authors:** Cosme Alvarado-Esquivel, Sergio Estrada-Martínez

**Affiliations:** 1 Faculty of Medicine and Nutrition, Juárez University of Durango State. Avenida Universidad S/N. 34000 Durango, Durango, Mexico; 2 Institute for Scientific Research, Juárez University of Durango State. Avenida Universidad S/N. 34000 Durango, Durango. Mexico

## Abstract

**Background:**

We performed a retrospective, observational study in 1156 adult subjects from the general population of Durango City, Mexico, Fifty five subjects with a history of abdominal hernia repair and 1101 subjects without hernia were examined with enzyme-linked immunoassays for the presence of anti-*Toxoplasma *IgG and IgM antibodies.

**Results:**

The seroprevalence of anti-*Toxoplasma *IgG antibodies and IgG titers was significantly higher in subjects with abdominal hernia repair than those without hernia. There was a tendency for subjects with hernia repair to have a higher seroprevalence of anti-*Toxoplasma *IgM antibodies than subjects without hernia. The seroprevalence of anti-*Toxoplasma *IgG antibodies in subjects with hernia repair was significantly higher in subjects ≥ 50 years old than those < 50 years old. Further analysis in subjects aged ≥ 50 years showed that the seroprevalence of anti-*Toxoplasma *IgG antibodies was also significantly higher in individuals with hernia repair than those without hernia (OR = 2.72; 95% CI: 1.10-6.57). Matching by age and sex further showed that the seroprevalence of *Toxoplasma *infection was significantly higher in patients with hernia repair than those without hernia (OR: 4.50; 95% CI: 1.22-17.33).

**Conclusions:**

Results indicate that infection with *Toxoplasma *is associated with abdominal hernia. The contributing role of infection with *Toxoplasma *in abdominal hernia was observed mainly in subjects aged ≥ 50 years old. Our results might have clinical, prevention and treatment implications and warrant for further investigation.

## Findings

The protozoan parasite *Toxoplasma gondii *(*T. gondii*) is widely distributed around the world [1,2]. Human infections with *T. gondii *occur by ingesting food or water that is contaminated with oocysts shed by cats or by eating undercooked or raw meat containing tissue cysts [2-4]. Infections with *T. gondii *may result in an asymptomatic state or lead to disease. The parasite disseminates within the host's body and may affect lymph nodes, eyes, central nervous system, and other tissues [3,5-9]. In addition, primary infection during pregnancy may lead to severe damage to the fetus [2,3]. We have explored the seroprevalence of and risk factors for *T. gondii *infection in some healthy [10-12] and ill [13-16] populations in Durango, Mexico. In a recent study in liver disease patients, we reported that subjects with abdominal hernia repair showed a significantly higher seroprevalence of *T. gondii *infection than individuals without hernia [16]. Therefore, we sought to determine whether the seroprevalence of *T. gondii *infection and anti-*T. gondii *IgG levels are associated with a history of abdominal hernia repair in subjects of the general population in Durango, Mexico. Furthermore, we investigated socio-demographic, clinical, and behavioral characteristics associated with *T. gondii *seropositivity in subjects with abdominal hernia repair.

Through a retrospective, observational study design, we studied 1156 subjects of the general population of Durango City, Mexico that were examined for *T. gondii *antibodies in our Faculty of Medicine from January 2009 to December 2010. Of the 1156 subjects, 55 had a history of abdominal hernia repair while 1101 subjects did not report any suffering from abdominal hernia or having had any abdominal hernia repair.

Socio-demographic, clinical and behavioral characteristics of the participants were obtained with the aid of a standardized questionnaire and kept in records. Socio-demographic data included age, gender, place of birth, place of residence, residence area (urban, suburban, rural), educational level, socioeconomic status, and occupation. Clinical data included the presence of diseases, presence or history of lymphadenopathy, frequent presence of headache; history of blood transfusion, transplant, or surgery; and memory, reflex, hearing, and visual impairments. Behavioral data included animal contacts, contact with cat feces, traveling in Mexico and abroad, meat consumption (pork, beef, goat, sheep, boar, chicken, turkey, pigeon, rabbit, venison, squirrel, horse, opossum, or other), degree of meat cooking, consumption of unpasteurized milk, dried or cured meat (ham, sausages, salami or chorizo), unwashed raw vegetables, fruits, or untreated water, frequency of eating out of home (at restaurants or fast food outlets), contact with soil (gardening or agriculture), and type of floors at home.

Sera were analyzed by qualitative and quantitative methods for anti-*T. gondii *IgG antibodies with the commercially available enzyme immunoassay kit "*Toxoplasma *IgG" (International Immuno-Diagnostics, Foster City, California). Anti-*T. gondii *IgG antibody levels were expressed as International Units (IU)/ml, and a result equal or greater than 8 IU/ml was considered positive. In addition, sera positive for anti-*T. gondii *IgG antibodies were further analyzed for anti-*T. gondii *IgM antibodies by the commercially available enzyme immunoassay "*Toxoplasma *IgM" kit (International Immuno-Diagnostics). All tests were performed following the instructions of the manufacturer.

This study was approved by the Institutional Ethical Committee of the Institute of Security and Social Services of the State Workers in Durango City.

Results were analyzed with the aid of the software Epi Info version 3.5.1 and SPSS 15.0 (SPSS Inc. Chicago, Illinois). For comparison of the frequencies among groups, the Fisher exact test was used. A bivariate analysis was used to assess the association between subject's characteristics and *T. gondii *infection. Odds ratio (OR) and 95% confidence interval (CI) were calculated to assess associations. A *P *value less than 0.05 was considered statistically significant.

Anti-*T. gondii *IgG antibodies were found in 9 (16.4%) of 55 subjects with hernia repair and in 76 (6.9%) of 1101 subjects without hernia (OR = 2.64; 95% CI: 1.16-5.85; *P *= 0.01). The seroprevalence of *T. gondii *infection and the socio-demographic characteristics of the subjects with hernia repair are shown in Table 1. Seroprevalence of *T. gondii *infection in subjects with hernia repair was significantly higher in individuals who were not born in Durango State than those born in Durango State (*P *= 0.03), and in individuals of medium socioeconomic level than those of low socioeconomic level (*P *= 0.04). Anti-*T. gondii *IgG levels were significantly higher in subjects with hernia repair than those without hernia (*P *= 0.03) (Table 2). Anti-*T. gondii *IgM antibodies were found in 4 (7.3%) subjects with hernia repair and in 27 (2.5%) subjects without hernia (*P *= 0.05). An age-stratified seroprevalence of *T. gondii *infection is shown in Table 3. Seroprevalence of anti-*T. gondii *IgG antibodies in hernia subjects was significantly higher in subjects ≥ 50 years old (9/36: 26.7%) than those < 50 years old (0/18: 0%) (*P *= 0.02). Since *T. gondii *infection in subjects with hernia repair was found only in individuals aged ≥ 50 years old, we used this age group for further comparison. Seroprevalence of anti-*T. gondii *IgG antibodies in subjects aged ≥ 50 years old was significantly higher in individuals with hernia repair (9/36: 25%) than those without hernia (42/385: 10.9%) (OR = 2.72; 95% CI: 1.10-6.57; *P *= 0.02). We further matched these subjects (≥ 50 years old) with a history of hernia with those without any history of hernia by age and sex. We analyzed two controls for each case, and the seroprevalence of *T. gondii *infection was significantly higher in patients with a history of hernia (9/35: 25.7%) than those without any history of hernia (5/70: 7.1%) (OR: 4.50; 95% CI: 1.22-17.33).

**Table 1:** Socio-demographic characteristics of subjects with abdominal hernia repair and seroprevalence of *T. gondii *infection.

			Prevalence of *T. gondii *infection		*P *value
				
Characteristic	No.^a^	*%*	**No**.	*%*	
Age groups (years)					
30 or less	7	13	0	0	0.06
31-50	11	20.4	0	0	
> 50	36	66.7	9	100	
Sex					
Male	32	58.2	3	9.4	0.14
Female	23	41.8	6	26.1	
Birth place					
Durango State	45	83.3	5	11.1	0.03
Other Mexican State	9	16.7	4	44.4	
Residence place					
Durango State	55	100	9	16.4	-
Residence area					
Urban	48	88.9	9	18.8	0.50
Suburban	2	3.7	0	0.0	
Rural	4	7.4	0	0.0	
Educational level					
No education	1	1.9	0	0.0	0.67
1-6 years	19	35.2	4	21.1	
7-12 years	14	25.9	1	7.1	
> 12 years	20	37.0	4	20.0	
Socio-economic level					
Low	15	28.3	0	0.0	0.04
Medium	38	71.7	9	23.7	
Occupation					
Non-laborer^b^	15	27.3	2	13.3	1.00
Laborer^c^	40	72.7	7	17.5	

^a^In some strata the sum does not add up to the total because of a few missing values.

^b^Non laborer = none occupation, student or housewife.

^c^Laborer = Employee, professional, business, agriculture, cattle rising, factory worker, construction worker or other.

**Table 2:** Comparison of anti-*Toxoplasma *IgG levels in subjects with hernia repair and subjects without hernia.

Anti-*Toxoplasma *IgG levels	Subjects with hernia repair (n = 55)		Subjects without hernia (n = 1101)		*P *value
		
	**No**.	*%*	**No**.	*%*	
< 90 IU/ml	2	3.6	18	1.6	0.25
≥ 90 IU/ml	7	12.7	58	5.3	0.03

**Table 3:** Seroprevalence of anti-*T. gondii *IgG antibodies in subjects with hernia repair and subjects without hernia according to age groups.

Age groups (years)	Subjects with hernia repair			Subjects without hernia				
			
	**No**.	*T. gondii *infection		**No**.	*T. gondii *infection		Odds ratio	*P *value
	tested	**No**.	%	tested	**No**.	%	(95% Confidence interval)	
≤ 29	7	0	0.0	403	15	3.7	0.00 (0.00-19.66)	1.00
30-39	7	0	0.0	184	15	8.2	0.00 (0.00-8.62)	1.00
40-49	4	0	0.0	122	4	3.3	0.00 (0.00-59.51)	1.00
50-59	6	1	16.7	153	14	9.2	1.99 (0.04-19.58)	0.45
60-69	15	4	26.7	115	13	11.3	2.85 (0.57-11.50)	0.11
≥ 70	15	4	26.7	117	15	12.8	2.47 (0.50-9.76)	0.23
								
≥ 50	36	9	25.0	385	42	10.9	2.72 (1.10-6.57)	0.02
								
All	54^a^	9	16.7	1094^a^	76	6.9	2.68 (1.17-5.95)	0.01

^a^Subjects with available age.

None of the clinical characteristics including the presence of underlying diseases, presence or history of lymphadenopathy, frequent presence of headache; history of blood transfusion, transplant, or other surgeries; and memory, reflex, hearing, and visual impairments in subjects with hernia repair were associated with *T. gondii *seropositivity. Concerning behavioral characteristics, the bivariate analysis showed that *T. gondii *infection was negatively associated with the variable untreated water consumption (*P *= 0.02). Other behavioral characteristics in the subjects with hernia repair did not show any association with *T. gondii *infection.

In this study, we found a significantly higher seroprevalence of anti-*T. gondii *IgG antibodies in subjects with a history of abdominal hernia than subjects without history of hernia. Anti-*T. gondii *IgG antibody levels were also significantly higher in subjects with a history of hernia than subjects without this history. Prevalence of anti-*T. gondii *IgM antibodies was higher in subjects with hernia repair than those without hernia, and this difference in seroprevalences among the groups showed a borderline statistical significance. Results indicate a stronger seropositivity to *T. gondii *in subjects with a history of abdominal hernia than subjects without hernia. The association of *T. gondii *infection and abdominal hernia was found in the whole population studied and especially in subjects aged ≥ 50 years old. Overall, the prevalence of hernia among the general population studied was low (4.8%). Therefore, the hernia group was small compared with the much larger control group. However, a reduction of the control group size by age and sex matching (two controls for every case) confirmed our results and even increased the odd ratio. It is not clear why the seroprevalence of *T. gondii *infection was higher in abdominal hernia patients than in subjects without hernia. Transmission of *T. gondii *infection by surgical procedures other than transplantation is not currently acknowledged. In a previous study in psychiatric patients, we found that patients with a history of surgery had a significantly higher seroprevalence of *T. gondii *infection than patients without this history [13]. In the present study, transmission of infection by the surgical procedure during hernia repair cannot be ruled out. However, this route of infection seems unlikely since some subjects without hernia repair has had other surgical procedures too and their seroprevalence was lower than those with hernia repair. On the other hand, there is not any published data concerning a role of *T. gondii *infection in the pathogenesis of abdominal hernia. Alterations in skeletal muscles of the abdominal wall have been involved in the pathogenesis of abdominal hernia; these alterations include disruption of muscles [17], and degenerative changes in muscle fibers [18]. The parasite *T. gondii *exists in skeletal muscles of infected humans and animals [2,19,20], and viable *T. gondii *can be isolated from animal muscular tissues [21]. In humans, infections with *T. gondii *may cause muscle disease (myositis or polymyositis) that may lead to myalgias and muscular weakness [22-26]. In mice experimentally infected with *T. gondii*, severity of muscle alterations depended upon concentration of parasites [20]. Therefore, *T. gondii *might contribute in the pathogenesis of abdominal hernia in some individuals by affecting their abdominal muscles.

Why high anti-*T. gondii *IgG levels were more frequency observed in subjects with hernia repair than in subjects without hernia is not clear. It is likely that a continuous parasite antigenic stimulation may induce a high antibody production in the hosts. On the other hand, the stress of the surgical procedure might have reactivated latent *T. gondi *infections leading to an increase of anti-*T. gondii *IgG antibodies. Surgical procedures may cause immunodepression [27-29], and reactivations of viral and parasitic infections after surgery have been reported in humans and animals [30-33].

In the present study with an independent population, we confirmed our previous report of an association of *T. gondii *infection with abdominal hernia [16]. Our results might have clinical, prevention and treatment implications. The results warrant for further investigation.

## Competing interests

The authors declare that they have no competing interests.

## Authors' contributions

CAE conceived and designed the study protocol, participated in the coordination and management of the study, applied the questionnaires, performed the laboratory tests, the data analysis and statistical analysis, and wrote the manuscript. SEM performed the statistical analysis. Both authors approved the final version of the manuscript.

## Funding

This study was supported by The Faculty of Medicine and Nutrition. Universidad Juárez del Estado de Durango. Durango City, Mexico.
